# Synthesis of Large-Area Single-Layer Graphene Using Refined Cooking Palm Oil on Copper Substrate by Spray Injector-Assisted CVD

**DOI:** 10.1186/s11671-019-2976-0

**Published:** 2019-04-24

**Authors:** Saleha Maarof, Amgad Ahmed Ali, Abdul Manaf Hashim

**Affiliations:** Malaysia-Japan International Institute of Technology, Universiti Teknologi Malaysia, Jalan Sultan Yahya Petra, 54100 Kuala Lumpur, Malaysia

**Keywords:** Nanocarbon, Graphene, Green synthesis, Copper substrate, Natural precursor, Spray-assisted CVD

## Abstract

We present a synthesis of large-area single-layer graphene on copper substrate using a refined cooking palm oil, a natural single carbon source, by a home-made spray injector-assisted chemical vapor deposition system. The effects of the distance between spray nozzle and substrate, and growth temperature are studied. From Raman mapping analysis, shorter distance of 1 cm and temperature of around 950 °C lead to the growth of large-area single-layer graphene with a coverage up to 97% of the measured area size of 6400 μm^2^. The crystallinity of the grown single layer graphene is relatively good due to high distribution percentage of FWHM values of 2D band that is below 30 cm^−1^. However, the defect concentration is relatively high, and it suggests that a flash-cooling technique needs to be introduced.

## Introduction

Graphene, a two-dimensional nanomaterial, possesses a *sp*^*2*^-hybridized carbon atom bonding with single atom thick [1]. Its extraordinary properties such as superior electronic transport, thermal conductivity, mechanical durability, and so forth have attracted tremendous studies for various potential applications in nanoelectronics [2], optoelectronics [3], super capacitors and electrochemical energy storages [4], solar cells [5], and sensors [6]. In fact, many applications such as wearable detectors, electronic skin, and pressure sensors require flexible large-area graphene structures [7]. Thus, in order to bring graphene into practical applications, a technology to realize large-area graphene with uniform thickness and defect-free is absolutely demanded. Since micromechanical exfoliation seems to have a limitation in obtaining large-area graphene with uniform thickness even though it can produce high crystalline graphene with less defect [8, 9], chemical vapor deposition (CVD) has been considered as a promising technique to overcome such limitation [10, 11]. In principle, the quality of CVD-grown graphene is controlled by several main growth parameters, such as carbon source, temperature, substrate, and pressure [12]. Generally, it requires elevated temperature (greater than 800 °C) to grow high-quality graphene by CVD. However, a modified CVD process, specifically carbon-enclosed CVD (CE-CVD) method, was reported to be able to grow graphene onto Cu foil at a low temperature of nearly 500 °C [13]. In CVD technique, typically, graphene is grown on metal substrate using toxic and explosive hydrocarbon gases such as methane [14], acetylene [15], and propylene [16] via low-pressure [17] or atmospheric-pressure CVD [18], which lead to the use of the growth systems with high degree of safety and handling precautions.

Many benign alternative attempts have been made to replace these typical precursors with moderate hazardous hydrocarbon supplied from liquid or solid carbon sources. For an example, Weiss et al. investigated the growth of graphene on copper (Cu) substrate by utilizing ethanol [19]. Choi et al. reported the growth in oxidized ambient by using a combination of ethanol and methanol as a carbon source [20]. Other similar liquid carbon sources such as benzene [21] and toluene [22] have also being studied. A motivated result on the growth of graphene from natural carbon sources such as camphor [23, 24] has also being reported. Recently, we have reported the growth of defect-free mixed single and bi-layer graphene on nickel (Ni) substrate using a refined cooking palm oil [25, 26] by thermal CVD. Here, the evaporated refined cooking palm oil was delivered to the Ni substrate by constant flow of the argon/hydrogen (Ar/H_2_) carrier gas. The growth was performed at temperature of 900 °C for 15 s, before it was rapidly cooled down by the flash-cooling technique. However, the coverage of the grown graphene is relatively low of around 60%. In this paper, we demonstrate an alternative route to synthesize large-area single-layer graphene with the coverage up to 97% utilizing a homemade spray injector-assisted CVD system without introducing H_2_ during the growth for the first time. This spray injector enables the atomization of precursor into micron-sized droplets. The atomized droplets enable better decomposition kinetics due to the increase of surface compared to conventional CVD methods. One more privilege is that the precursor injection flow rate allows the control of the droplet flux which controls the mass transfer rate during the vapor deposition [27].

## Methods

**Fig. 1:**
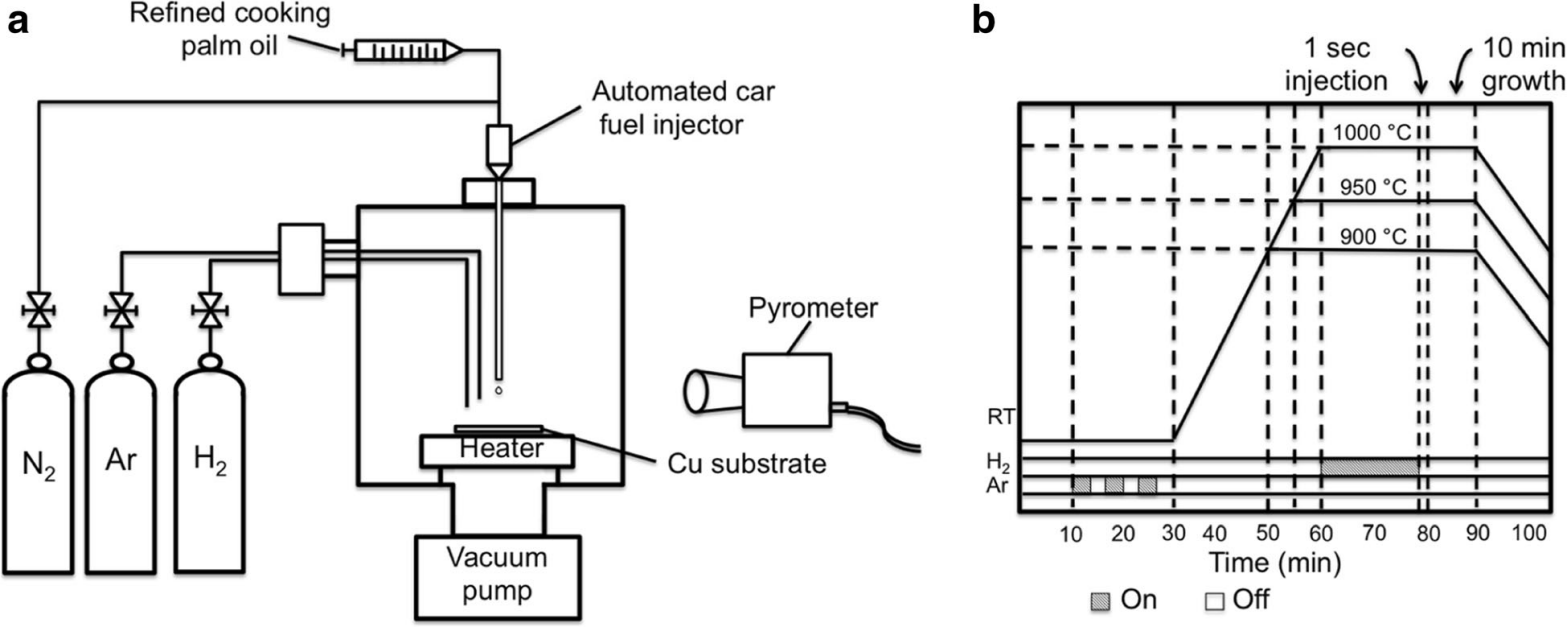
**a** Schematic of a homemade spray injector-assisted CVD setup and **b** growth time chart

A commercial Cu foil (Nilaco, 99.9% purity, 30 μm thick) is used as a metal catalyst. Firstly, a Cu foil cut into 1 cm × 1 cm is rinsed with distilled (DI) water, followed by a treatment using 1 M acetic acid/H_2_O (1:10) at 60 °C for 30 min. Then, this Cu sample is rinsed with isopropyl alcohol and acetone for 10 min in an ultrasonic bath (35% of power, UP400S, Hielscher, Germany) to remove any contamination and native oxide from the surface. Then, the Cu sample is dried using a nitrogen blow. Figure 1a and b show the schematic of a homemade spray injector-assisted CVD setup and the growth time chart, respectively. A specific amount of liquid refined cooking palm oil is delivered into the chamber by a high precision fluid injection system (Sono-Tek, USA) with an injection capability of 0.01 ml/s. A treated Cu substrate is then loaded into the reaction chamber facilitated with substrate heater as shown in Fig. 1a. After loading the Cu substrate, the reaction chamber is evacuated by a rotary pump down to 6 Pa before being purged with Ar. These evacuations and Ar purging processes are repeated for three times to minimize the trapped air in the reaction chamber.

The effects of the distance between the nozzle and the substrate, *d*, are studied. Here, *d* is set at 1, 3, and 6 cm. The substrate is heated to the set growth temperatures, *T*, i.e., 900, 950, and 1000 °C while keeping the reaction chamber in Ar environment. After reaching the set temperature, hydrogen (H_2_) of 40 sccm is introduced for 20 min. This annealing treatment in H_2_ is performed with the purpose to further remove the remaining contamination and to reduce the roughness of the Cu surface. After that, the flow of H_2_ is stopped and a refined cooking palm oil is injected for 1 s (~ 0.05 ml) into the reaction chamber using a computerized fuel injector (car fuel injector). Then, the growth (or heating) is kept at the set temperature for 10 min. After the growth, the heater is turned off and the sample is cooled down to a room temperature in vacuum ambient with continuous evacuation. Since an automated spray injector is used in this work to control the carbon (C) amount or concentration, it is expected that the C element should be able to reach and spread uniformly on the heated substrate after the effective thermal decomposition of refined cooking palm oil. The decomposition can be expressed by the following reaction:

1 $$ {\mathrm{CH}}_3{\left({\mathrm{CH}}_2\right)}_{14}\mathrm{COOH}\to 16\mathrm{C}+16{\mathrm{H}}_2\uparrow +{\mathrm{O}}_2\uparrow $$

The growth mechanism is assumed to follow the well-accepted mechanism described in [22, 23]. Here, the decomposed C element is absorbed into the Cu substrate during the heating stage and then is desorbed back to the surface of Cu substrate to form graphene layer during the cooling stage. Since the cooling is carried by the continuous evacuation, it is speculated that the substrate is cooled down at relatively faster speed.

The optical microscopy is used to observe the morphology and homogeneity of the as-grown graphene films on Cu substrate. The structural characteristic, such as the number of graphene layers, homogeneity, and defects, are examined using micro-Raman spectroscopy (WiTec Alpha 300) at an excitation laser wavelength of 514 nm. Here, a × 100 magnification lens is used, giving a laser spot size of about 400 nm. The time integration is 0.5 s, and the laser power is kept below 1 mW to avoid any damage or heating on the sample, which may induce the desorption of the adatoms from graphene. The spectrometer is equipped with a piezoelectric stage that allows Raman mapping of an area up to 200 μm × 200 μm. To investigate the inhomogeneity of the graphene film, Raman mapping is used to collect a large quantity of spectra with different amount of disorder. Here, the analyzed number of spectra is 1024 for the size of 80 × 80 μm. Raman measurements are done without transferring the graphene film onto a new flat substrate. Hence, it can be said that the data presentation of graphene is in its original state. It is worth to note that the strong background signal from the Cu substrate has been removed from each spectrum by manual subtraction.

## Results and Discussion

**Fig. 2:**
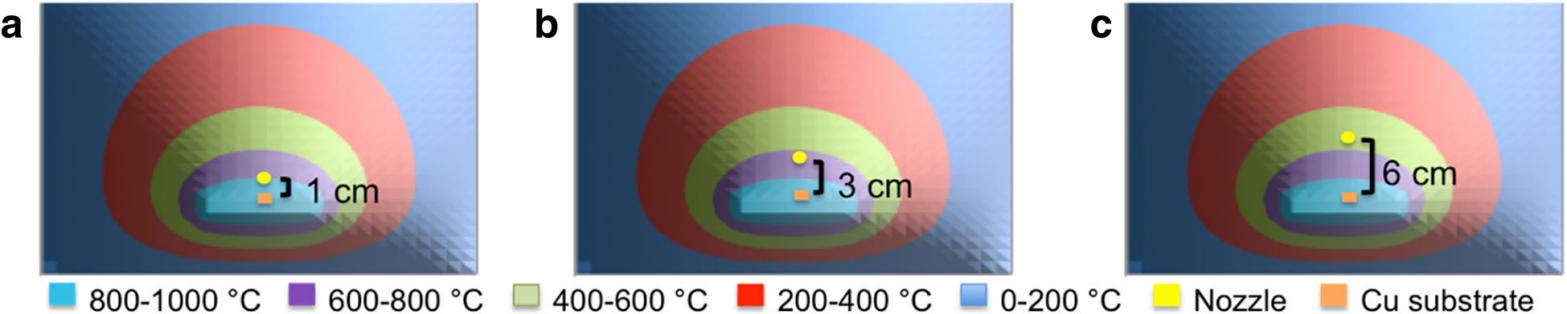
Heat distribution (cross-view) in the reaction chamber and the location of nozzle from the substrate with a distance of **a** 1 cm, **b** 2 cm, and, **c** 6 cm

Figure 2a–c show the simulated heat distribution (cross-view) in the reaction chamber together with the location of nozzle at the distance of 1, 3, and 6 cm from the substrate. Extending Fourier’s law to a two-dimensional vector quantity results in the heat flux per unit area as in Eq. 2, where the thermal conductivity relates the heat flux and the temperature gradient linearly. *q*_*xy*_ is the heat flux in the *x* and *y* directions (W/m^2^), *k* is the thermal conductivity constant (W/m K), and T is the temperature (K).

2 $$ {\overrightarrow{q}}_{xy}=-k\left(i\frac{\partial T}{\partial x}+j\frac{\partial T}{\partial y}\right) $$

A finite difference method was used to solve the equation. Thus, for the sake of differential control elements, only the factoring of steady-state conduction of energy conservation takes place as in Eq. 3.

3 $$ \frac{\partial }{\partial x}\left(k\frac{\partial T}{\partial x}\right)+\frac{\partial }{\partial y}\left(k\frac{\partial T}{\partial y}\right)+\dot{q}=0 $$

**Fig. 3:**
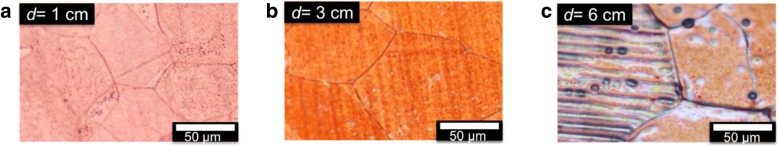
The optical images of the graphene on Cu substrate grown at temperature 1000 °C with the distance between a nozzle and a substrate of **a** 1 cm, **b** 3 cm, and **c** 6 cm growth

Respectively, when the substrate temperature is set in the range of 800–1000 °C. Such locations have been selected in this study so that the palm oil is injected from three different region of temperatures, i.e., 800–1000 °C (1 cm), 600–800 °C (3 cm), and 400–600 °C (6 cm). Figure 3a–c show the optical image of as-grown graphene on Cu at *d* = 1, 3, and 6 cm, respectively, grown at temperature of 1000 °C. It is well reported that higher temperature is better for the complete decomposition of source as well as for uniform absorption of C element into the Cu substrate. As can be seen in Fig. 3a, the color of Cu surface is almost similar to the original color of non-heated Cu substrate, indicating very few graphene layers. The color becomes slightly darker for the sample with *d* = 3 cm. However, many black spots are observed for the samples grown at *d* = 6 cm, indicating the formation of amorphous carbon in the cavities (holes), and scratches of Cu substrate [26]. It is worth noting that the treatment of metal substrate by H_2_ annealing can reduce the roughness of the surface [26]. However, such cavities and scratches which are generally formed during the production of Cu foil cannot be eliminated if the depth is too large. It has been reported that amorphous carbon is easy to be formed in such cavities and scratches due to the accumulation of C element. From these results, it can be assumed that *d* = 1 cm is the best distance to produce uniform few graphene layers with excellent suppression of amorphous carbon structures.

**Fig. 4:**
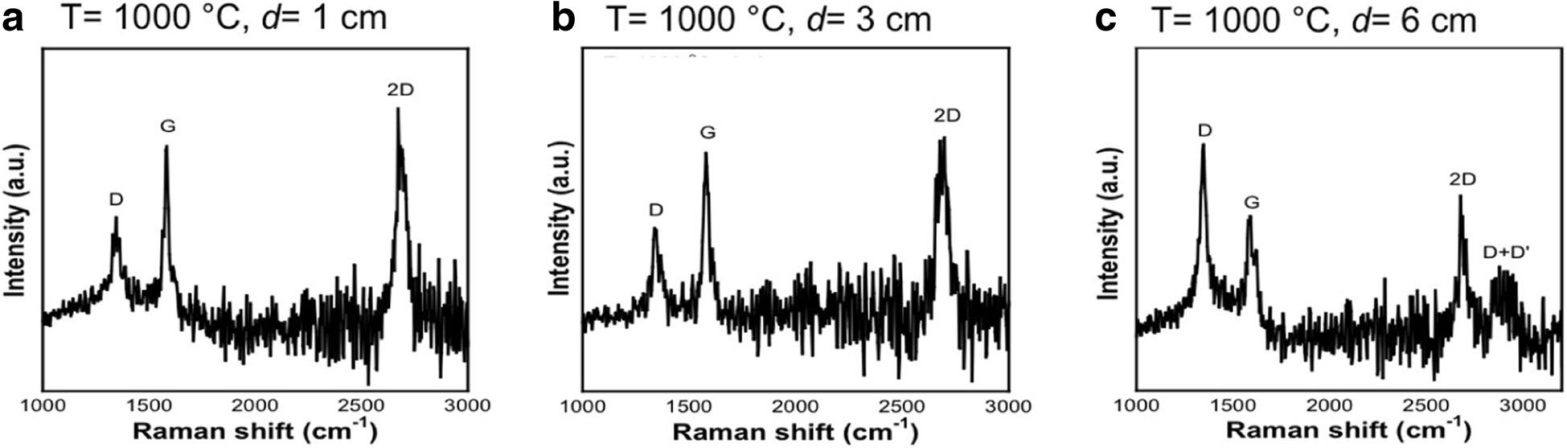
Single Raman spectrum of graphene on Cu substrate grown at temperature of 1000 °C with the distance between a nozzle and a substrate of **a** 1 cm, **b** 3 cm, and **c** 6 cm

**Fig. 5:**
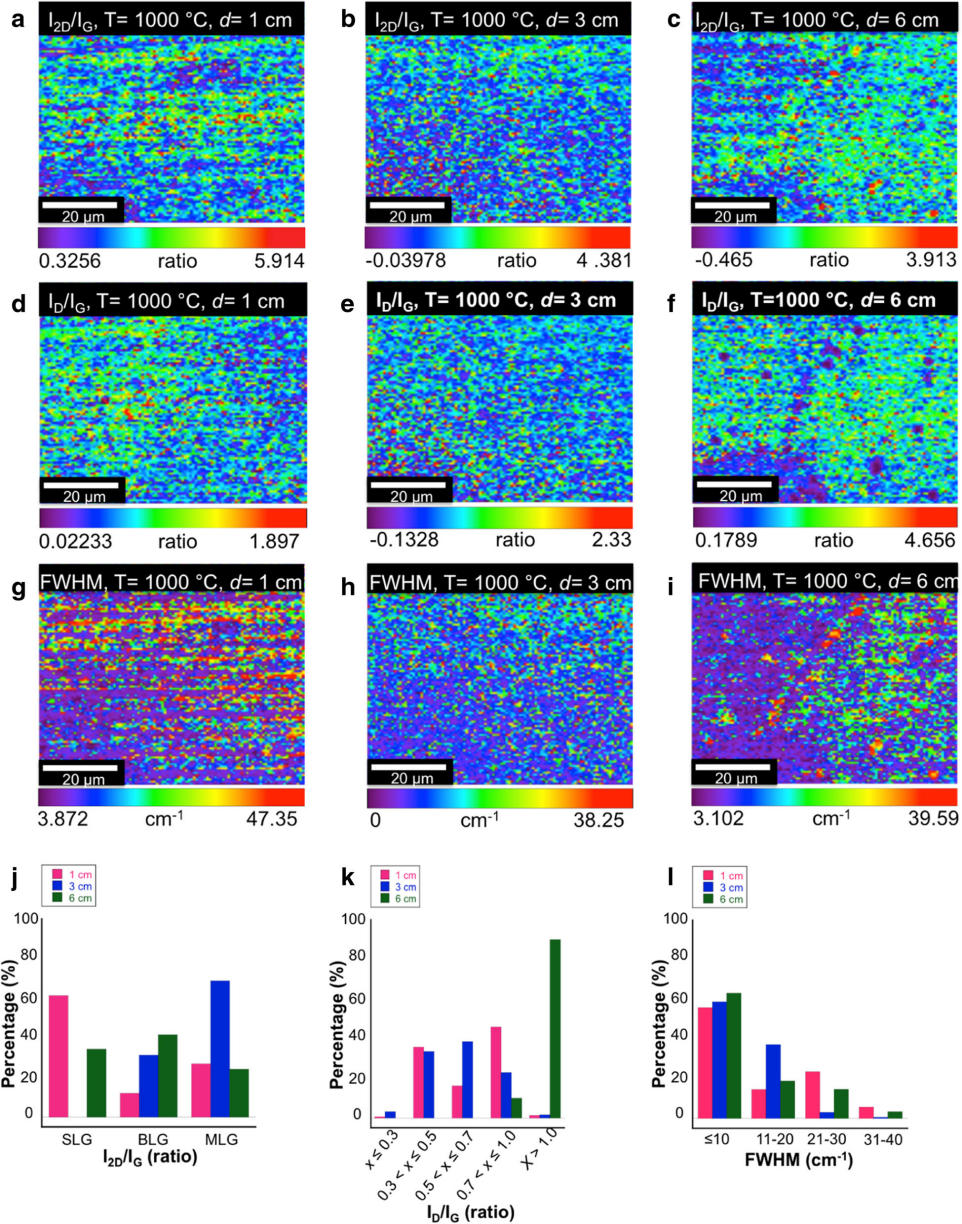
Raman mapping images of graphene on Cu substrate for **a**–**c** *I*_2D_/*I*_G_, **d**–**f** *I*_D_/*I*_G_, and **g**–**i** FWHM value of 2D band, for 1 cm, 3 cm, and 6 cm. Also, the histograms to indicate the distribution percentage of the intensity ratio, **j** *I*_2D_/*I*_G_, **k** *I*_D_/*I*_G_, and **l** FWHM value of 2D band. Growth temperature 1000 °C

Figure 4a–c show the Raman spectra of the graphene grown at *d* = 1, 3, and 6 cm, respectively. Three intense peaks at ~ 1350 cm^−1^, ~ 1560 cm^−1^, and ~ 2691 cm^−1^ corresponding to G, D, and 2D bands, respectively, can be clearly observed in all samples. A peak corresponded to D + D’ band (~ 3250 cm^−1^) is only observed in the sample grown at *d* = 6 cm indicating the existence of amorphous carbon in the structure, as shown in Fig. 4c. Figure 5a–c show the Raman mapping of the intensity ratio of 2D and G bands (*I*_2D_/*I*_G_), Fig. 5d–f the Raman mapping of the intensity ratio of D and G bands (*I*_D_/*I*_G_), and Fig. 5g–i the values of full-width half maximum (FWHM) of the 2D band for each distance, i.e., 1, 3, and 6 cm. Based on these Raman mapping, the histograms to indicate the distribution percentages of the *I*_2D_/*I*_G_, *I*_D_/*I*_G_, and FWHM are presented in Fig. 5j–l, respectively. As shown in Fig. 5j, the sample grown at *d* = 1 cm tends to be dominated by single-layer graphene, whereas the samples grown at *d* = 3 and 6 cm are dominated by bilayer and multilayer graphene. It is worth to note that the determination of layer thicknesses is made based on the following values: single layer, *I*_2D_/*I*_G_ ≥ 2; bilayer, 1 ≤ *I*_2D_/*I*_G_ < 2; and multilayer, *I*_2D_/*I*_G_ < 1 [28, 29]. The sample grown at *d* = 1 cm seems to generate less defect concentration as compared to the sample grown at *d* = 6 cm as can be understood from Fig. 5k. The FWHM values of 2D band for all samples are mainly below 10 cm^−1^ indicating relatively high crystallinity of the grown graphene as shown in Fig. 5l. It can be concluded that the distance between the nozzle and substrate should be small so that the droplets can be effectively decomposed before it reaches the Cu surface and uniformly absorbed into the Cu surface.

**Fig. 6:**
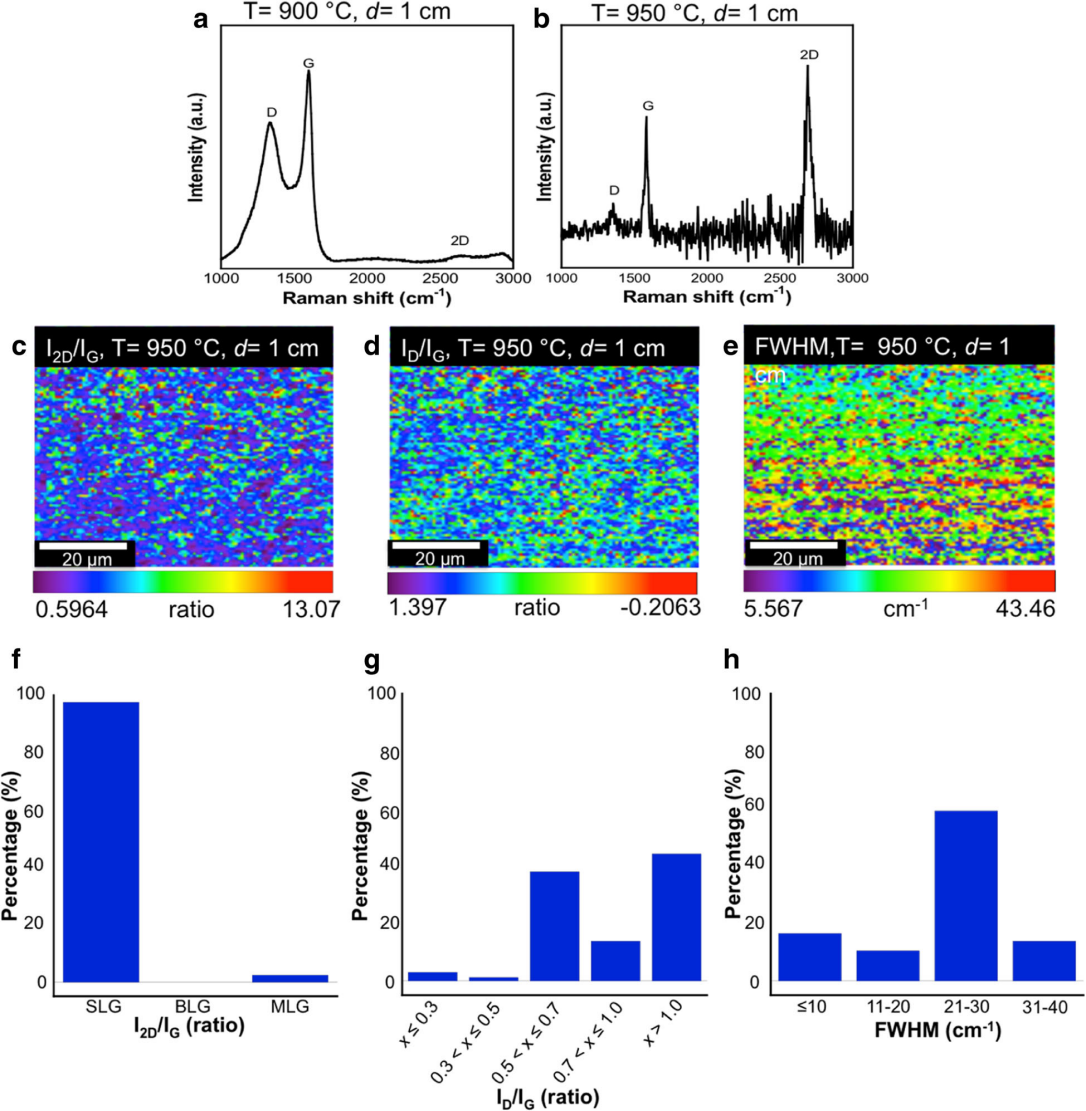
Single Raman spectrum for the sample grown at temperatures of **a** 900 °C and **b** 950 °C. Raman mapping images of sample grown on Cu substrate at temperature of 950 °C for **c** *I*_2D_/*I*_G_, **d** *I*_D_/*I*_G_, and **e** FWHM value of 2D band. Also, the histograms to indicate the distribution percentage of the intensity ratio, **f** *I*_2D_/*I*_G_, **g** *I*_D_/*I*_G_, and **h** FWHM value of 2D band. A distance between nozzle and substrate is 1 cm

Figure 6a and b show the Raman spectra of the samples grown at lower temperatures of 900 and 950 °C, respectively. Here, the distance between the nozzle and substrate is fixed at *d* = 1 cm since this distance is found to be a suitable distance to obtain the best quality of graphene. As shown in Fig. 6a, it can be said that the grown film at 900 °C is dominated by an amorphous carbon film and almost no graphene growth is observed. Meanwhile, the sample grown at 950 °C confirms the growth of graphene layer. Figure 6c–e show the Raman mapping of the *I*_2D_/*I*_G_, *I*_D_/*I*_G_, and FWHM of the 2D band for the sample grown at 950 °C, respectively. It clearly shows that the grown film possesses excellent layer uniformity by referring to the uniform color distribution. Histograms driven from these Raman mapping are used to indicate the distribution percentages of the *I*_2D_/*I*_G_, *I*_D_/*I*_G_, and FWHM as presented in Fig. 6f–h, respectively. As shown in Fig. 6f, the samples grown at such temperature seem to be dominated by single-layer graphene with a coverage up to 97%. However, the grown sample also seems to generate slightly higher defect concentration as compared to the sample grown at 1000 °C as can be understood by comparing Fig. 6g and Fig. 5k. This defect is speculated to be generated due to considerably slow rate of cooling. In this regards, a flash cooling was reported as a capable solution for obtaining a defect-free graphene layer. Graphene growth by CVD technique using Cu as a metal catalyst has been reported to exhibit a surface-mediated mechanism due to its low carbon solubility properties. Utilizing atmospheric pressure CVD (APCVD), large-area single-layer graphene is able to be grown. Unfortunately, under high carbon concentration, the decomposed C elements in the gas phase will keep depositing to form graphene stacking until the surface is covered by BLG and MLG. Here, the formation of graphene follows a segregation and precipitation of growth mechanism. Under such condition, a flash cooling is needed to suppress the graphene deposition. In addition, uniform graphene can be grown under low-pressure or ultrahigh vacuum condition CVD system. The rapid cooling results in reducing the size equiaxed Cu grains which will reduce the grain boundary sites. This will eventually force the redistribution of C atoms in a homogeneous uniform way. [25, 26]. The FWHM values of 2D band are mainly in the range of 21–30 cm^−1^ indicating relatively high crystallinity of the grown graphene as shown in Fig. 6h.

## Conclusions

A growth of large-area single-layer graphene on Cu substrate using a refined cooking palm oil, a natural single carbon source, by a home-made spray injector-assisted chemical vapor deposition system was performed. The effects of the distance between spray nozzle and substrate, and growth temperature are studied. The growth of large-area single-layer graphene with a coverage up to 97% of the measured area size of 6400 μm^2^ was obtained at optimum process conditions (growth temperature of 950 °C, and nozzle to substrate distance of 1 cm). The crystallinity of the grown single-layer graphene is relatively good with high distribution percentage of FWHM values of 2D band that is below 30 cm^−1^. However, the defect concentration is relatively high, and it suggests the requirement of a rapid cooling treatment. Further studies on the properties such as atomic structure, transmission, and resistance will further justify the performance of the present graphene as compared to the other grown graphene.

## Abbreviations

Ar: Argon
C: Carbon
Cu: Copper
CVD: Chemical vapor deposition
FWHM: Full-width half maximum
H_2_: Hydrogen
